# Reliability and Responsiveness of the Hand20 Questionnaire in Thumb Carpometacarpal Joint Osteoarthritis: A Pilot Study

**DOI:** 10.7759/cureus.83383

**Published:** 2025-05-03

**Authors:** Hirotomo Shibahashi, Kotaro Saka, Koichiro Abe, Kanta Ohno, Keizo Fukumoto

**Affiliations:** 1 Occupational Therapy, Department of Rehabilitation, School of Health Sciences, Tokyo University of Technology, Tokyo, JPN; 2 Department of Rehabilitation Medicine, Saitama Jikei Hospital, Saitama, JPN; 3 Saitama Hand and Microsurgery Institute, Saitama Jikei Hospital, Saitama, JPN

**Keywords:** hand20 questionnaire patient-reported outcome measures, patient reported outcome measures, reliability and responsiveness, thumb carpometacarpal joint osteoarthritis, upper extremity disorders

## Abstract

Background

Thumb carpometacarpal (CMC) joint osteoarthritis significantly impairs hand function and quality of life, particularly in older adults. Reliable patient-reported outcome measures (PROMs) are essential for assessing disease impact and evaluating treatment efficacy. The Hand20 questionnaire, widely used for upper extremity conditions, has demonstrated strong reliability and responsiveness. However, its specific utility in thumb CMC joint osteoarthritis remains unexplored.

Methods

This retrospective study included 20 patients diagnosed with thumb CMC joint osteoarthritis. Hand20 scores were collected at baseline, three months, and six months. Test-retest reliability was evaluated using the intraclass correlation coefficient (ICC) from a mixed-effects model. Responsiveness was assessed using the effect size (ES) and standardized response mean (SRM). Paired t-tests were used to analyze changes in scores over time.

Results

The mean Hand20 score decreased from 33.50 at baseline to 18.77 at three months and 15.93 at six months. The ICC was 0.672 (95% confidence interval {CI}: 0.406-0.823), indicating moderate-to-good reliability. Responsiveness was moderate at three months (ES: -0.650, SRM: -0.708) and large at six months (ES: -0.776, SRM: -0.940). Paired t-tests showed significant reductions in Hand20 scores over time, confirming progressive improvements ( *p *< 0.005).

Conclusion

The Hand20 exhibited moderate reliability and substantial responsiveness, underscoring its utility in evaluating treatment outcomes for thumb CMC joint osteoarthritis. Its ease of administration and sensitivity to clinical changes make it a valuable tool for clinicians. Further research with more diverse populations is needed to confirm these findings and explore their broader applicability.

## Introduction

Thumb carpometacarpal (CMC) joint osteoarthritis is a prevalent condition that substantially impairs hand function and quality of life, particularly in middle-aged and older adults [1,2]. Radiographic studies have reported that its prevalence increases with age, affecting approximately 15% of individuals over 30 and up to 33-39% in older women, with female sex and aging identified as significant risk factors [1,2]. This disease is characterized by pain, joint instability, and progressive loss of mobility, hindering daily activities such as pinching, gripping, and writing. Given the critical role of the thumb in hand function, reliable and responsive patient-reported outcome measures (PROMs) are essential for accurately assessing disease impact and evaluating the effectiveness of therapeutic interventions.

The Hand20 questionnaire is a widely used PROM for evaluating upper-extremity disabilities [3]. Designed as a simple and comprehensive tool for evaluating hand function, the Hand20 has demonstrated strong reliability, validity, and responsiveness across various upper-limb disorders [4]. While region-specific PROMs such as the Disabilities of the Arm, Shoulder, and Hand (DASH) questionnaire and generic health surveys like the 36-Item Short-Form Health Survey (SF-36) are frequently used, they may lack sensitivity to hand-specific impairments and have limited feasibility in elderly populations [5]. The Hand20, with its illustrated format and hand-focused content, addresses these limitations and has shown superior completion rates in older adults [3-5]. However, its specific utility in thumb CMC joint osteoarthritis has not been thoroughly examined. Thumb-specific impairments, such as difficulties with precision grip or thumb-index opposition, may not be fully captured by PROMs designed for broader upper-extremity conditions [5]. Thus, evaluating the reliability and responsiveness of Hand20 in this specific patient population is critical for establishing its clinical relevance.

This pilot study aimed to evaluate the test-retest reliability and responsiveness of the Hand20 questionnaire in patients with thumb CMC osteoarthritis. The goal was to determine whether Hand20 is a suitable PROM for assessing disease impact and monitoring clinical changes in this population. Findings from this preliminary investigation are intended to inform future large-scale studies and support the clinical utility of Hand20 in both research and practice.

## Materials and methods

Study sample

This retrospective study included 20 patients diagnosed with thumb CMC joint osteoarthritis between October 1, 2022, and October 31, 2023. All participants were evaluated and diagnosed by hand surgeons at Saitama Jikei Hospital and were prescribed hand therapy as part of their treatment. Data were collected at three time points: baseline (prior to treatment), three months, and six months after treatment. This study was conducted in accordance with the Declaration of Helsinki and approved by the Ethics Committee of the Tokyo University of Technology (approval number: E24HS-036, dated January 8, 2025). Written informed consent was obtained from all patients, and all data were anonymized to ensure patient confidentiality and privacy. All patients diagnosed with thumb CMC joint osteoarthritis and prescribed hand therapy during the study period were included. No exclusion criteria were applied. As this was a pilot study, no formal sample size calculation was performed. The sample size of 20 patients was pragmatically determined based on feasibility and the number of eligible patients available during the study period. Participants were enrolled using consecutive sampling from the outpatient population at the hand surgery department.

Hand20 questionnaire

The Hand20 questionnaire is the first PROM for upper limb disability developed in Japan. It incorporates simple text, illustrations, and a facial scale to facilitate understanding for children and older adults [4,5]. The Hand20 assesses not only the functional aspects of the elbow, forearm, wrist, and fingers but also includes items evaluating social concerns, such as appearance. The questionnaire comprises 20 items, each rated on a scale of 0-10. Scores were converted to a range of 0-100, with higher scores indicating greater disability. Participants with three or more missing responses were excluded from the analysis.

Reliability

Test-retest reliability was assessed using the intraclass correlation coefficient (ICC) derived from a linear mixed-effects model [6]. The model included random intercepts for individual patients to account for within-patient variability across different time points. All measurements were conducted under standardized conditions, including consistent instructions, environmental settings, and administration procedures. This approach allowed for the inclusion of hierarchical data structures and provided a robust estimate of the reliability. The confidence intervals (CIs) for the ICC were obtained through bootstrap resampling with 1,000 iterations. An ICC value ≥0.75 indicated good reliability [6]. Other forms of reliability, such as inter-rater reliability and internal consistency, were not assessed in this study. Inter-rater reliability was not applicable because the Hand20 is a self-administered questionnaire completed solely by the patient, without the involvement of external raters. Internal consistency, typically assessed using Cronbach’s alpha, was not recalculated because the Hand20 has previously demonstrated excellent internal consistency (α = 0.967-0.973) in earlier validation studies involving patients with upper extremity disorders​ [3,5]. The primary aim of the present study was to evaluate the temporal stability (test-retest reliability) of the Hand20 specifically in patients with thumb carpometacarpal joint osteoarthritis.

Responsiveness

Responsiveness to clinical changes was measured using effect size (ES) and standardized response mean (SRM) [6]. SRM was calculated as the mean change in the Hand20 scores divided by the standard deviation of the change scores. ES was calculated as the mean change in the Hand20 scores divided by the standard deviation of the baseline scores. The thresholds for interpreting ES and SRM were set as small (0.2), moderate (0.5), and large (0.8) [7].

Statistical analysis

Descriptive statistics were used to summarize the demographic and clinical characteristics of the participants. ICC values were computed to assess reliability, and paired t-tests were used to evaluate significant changes in the Hand20 scores before and after treatment. ES and SRM were calculated as described above. All analyses were conducted using R (version 4.4.1, R Foundation for Statistical Computing, Vienna, Austria), with statistical significance set at *p* < 0.05.

## Results

Baseline patient characteristics are summarized in Table 1. A total of 20 patients were included in this study, with a mean age of 60.55 ± 6.99 years. No patients were lost to follow-up, and all patients completed the final evaluation. Among the participants, 90% (18 patients) were women. Based on the Eaton classification, the majority of patients were categorized as stage 2 (50%), followed by stage 3 (35%), stage 4 (10%), and stage 1 (5%). The distribution of hand dominance was equal, with 50% right-handed and 50% left-handed. Hand20 scores progressively decreased over time, with mean scores of 33.50 ± 22.65 at baseline, 18.77 ± 21.24 at three months, and 15.93 ± 17.24 at six months.

**Table 1:** Demographic and clinical characteristics of patients SD: standard deviation

Variable	Value
Sex (female, %)	18 (90)
Age (mean, ±SD)	60.55±6.99
Eaton classification – Stage 1 (%)	1 (5)
Eaton classification – Stage 2 (%)	10 (50)
Eaton classification – Stage 3 (%)	7 (35)
Eaton classification – Stage 4 (%)	2 (10)
Dominant hand – Right (%)	10 (50)
Dominant hand – Left (%)	10 (50)
Hand20 – Baseline (mean, ±SD)	33.50±22.65
Hand20 – 3 months (mean, ±SD)	18.77±21.24
Hand20 – 6 months (mean, ±SD)	15.93±17.24

Reliability

The reliability of the Hand20 questionnaire was assessed using ICC. A mixed-effects model incorporating random intercepts for each patient yielded an ICC of 0.672 (95% CI: 0.406-0.823), indicating moderate-to-good reliability in patients with thumb CMC joint osteoarthritis. This model accounted for both within-patient variability and differences across time points, providing a robust estimate of reliability. Detailed results are shown in Table 2.

**Table 2:** Reliability and responsiveness of the Hand20 questionnaire ICC: intraclass correlation coefficient; ES: effect size; SRM: standardized response mean; CI: confidence interval

Index	Value
ICC	0.672 (95% CI: 0.406–0.823)
ES (Baseline–3 months)	-0.65
ES (Baseline–6 months)	-0.776
SRM (Baseline–3 months)	-0.708
SRM (Baseline–6 months)	-0.94

Responsiveness

**Figure 1:**
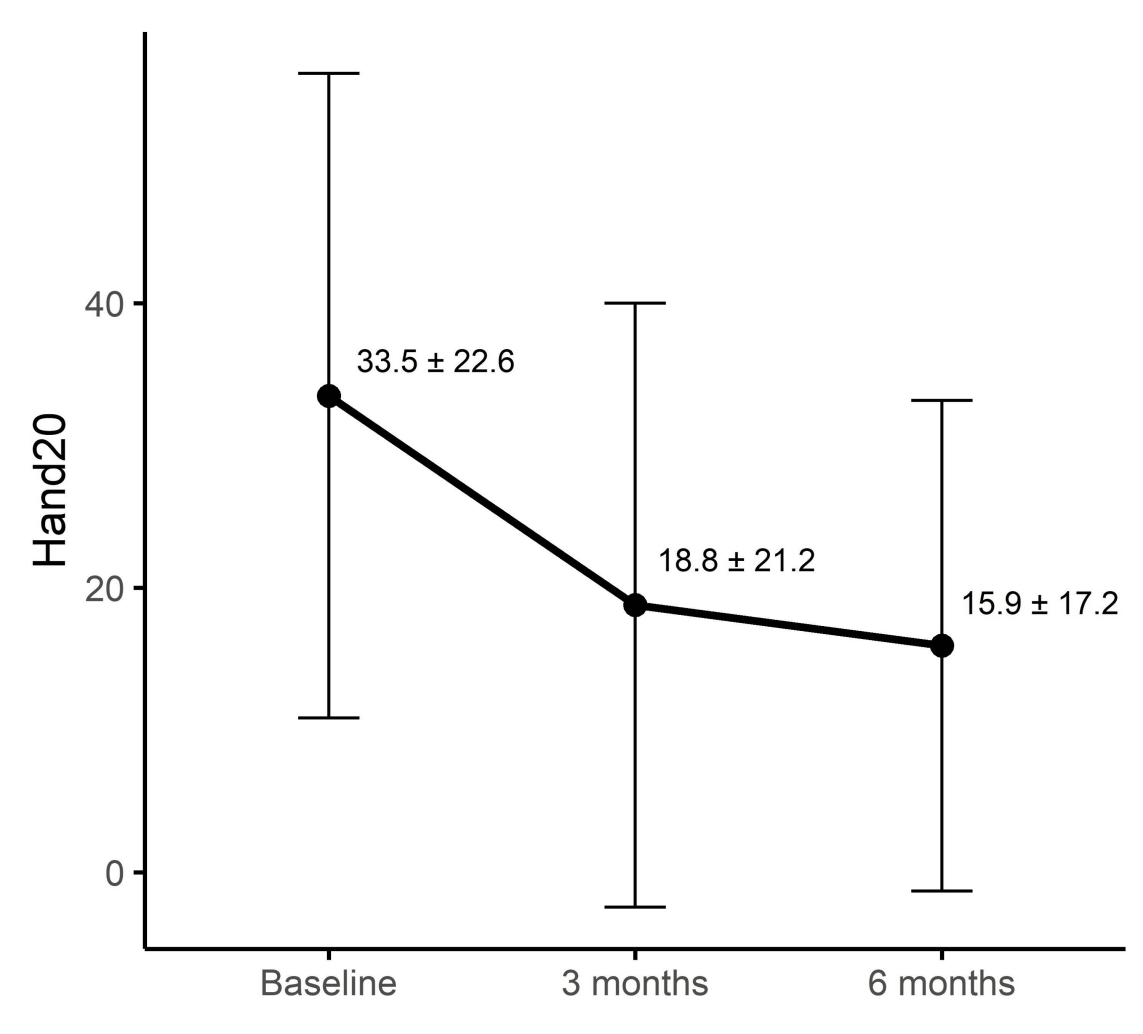
Time course of Hand20 scores at baseline, three months, and six months Mean Hand20 scores at baseline, three months, and six months are presented, with error bars representing standard deviations. The observed progressive decline in scores suggests sustained clinical improvement over the follow-up period.

The responsiveness of Hand20 was evaluated using ES and SRM. The ES from baseline to three months was -0.650, indicating moderate responsiveness, and increased to -0.776 at six months, approaching large responsiveness. Similarly, SRM was -0.708 at three months and -0.940 at six months, demonstrating moderate responsiveness at three months and large responsiveness at six months. These results indicate that Hand20 is sensitive to clinical changes over time, particularly over longer follow-up periods. The results are summarized in Table 2 and visually illustrated in Figure 1, where a progressive reduction in Hand20 scores can be observed across the follow-up period.

Statistical significance

Paired t-tests demonstrated significant improvements in the Hand20 scores over time. From baseline to three months, the mean difference was 14.73 (95% CI: 4.99-24.46; t = 3.167, p = 0.005), indicating a statistically significant reduction in scores. From baseline to six months, the mean difference increased to 17.58 (95% CI: 8.82-26.33; t = 4.202, *p* < 0.001), reflecting further improvements in hand function and symptom severity. These findings confirm significant and progressive reductions in the Hand20 scores over the treatment period. Comparative results are provided in Table 3.

**Table 3:** Paired comparisons of Hand20 scores over time CI: confidence interval

Comparison	Mean difference	95% CI	t-value	*p*-value
Baseline vs. 3 months	14.73	4.99 – 24.46	3.167	0.005
Baseline vs. 6 months	17.58	8.82 – 26.33	4.202	<0.001

## Discussion

This study evaluated the reliability and responsiveness of the Hand20 questionnaire in patients with thumb CMC joint osteoarthritis, addressing a gap in the literature regarding its utility in this specific population. The findings indicate that the Hand20 demonstrates moderate reliability and substantial responsiveness to clinical changes, supporting its potential as a valuable tool for assessing patient-reported outcomes in this context.

Reliability analysis of the Hand20 questionnaire revealed an ICC of 0.672 (95% CI: 0.406-0.823) using a mixed-effects model with random intercepts for each patient. This result aligns with the findings of a previous study that reported an ICC of 0.943 (95% CI: 0.931-0.952) for the Hand20 questionnaire in a different patient population [3], indicating moderate-to-good reliability in patients with thumb CMC joint osteoarthritis [8]. Moreover, the results showed ICC values comparable to those observed for other Japanese upper extremity PROMs, further underscoring the reliability of the Hand20 questionnaire [9]. By accounting for both within-patient variability and differences across time points, the mixed-effects model demonstrated its robustness in estimating reliability. These findings suggest that the Hand20 provides a stable and consistent measure of patient-reported outcomes, highlighting its utility in clinical settings for evaluating the impact of thumb CMC joint osteoarthritis. Additionally, the model's consideration of patient-specific variability emphasizes the importance of employing robust statistical methods when assessing the reliability of PROMs [7].

Responsiveness, a critical attribute of PROMs, was demonstrated using both ES and SRM. The Hand20 exhibited moderate responsiveness at three months (ES: -0.650, SRM: -0.708) and large responsiveness at six months (ES: -0.776, SRM: -0.940), demonstrating its ability to detect clinically meaningful changes over time. These findings are consistent with established interpretive benchmarks, wherein ES and SRM values of 0.50 and 0.80 are typically regarded as indicative of moderate and large responsiveness, respectively [6,10]. This pattern aligns with previous reports in which the Hand20 yielded ES and SRM values of -0.54 and -0.66, respectively, in patients with upper extremity conditions [3]. The observed increase in responsiveness over a longer follow-up period corresponds with expected treatment trajectories, as improvements in pain, mobility, and function often require sustained intervention. Furthermore, the Hand10 questionnaire, a shorter version of the Hand20, demonstrated similarly large responsiveness, with ES and SRM values of 1.38 and 1.18, respectively, in assessing lateral epicondylitis [11]. The robust SRM value at six months underscores the Hand20's heightened sensitivity in capturing these changes, reaffirming its utility for monitoring long-term outcomes. In addition, a cross-cultural adaptation of the Hand20 for Greek patients reported satisfactory responsiveness, indicating its potential for use in diverse populations and clinical settings [12]. In a similar context, the Thumb Disability Examination (TDX), a disease-specific PROM developed for thumb CMC osteoarthritis, has shown high concurrent validity with the Brief Michigan Hand Questionnaire (bMHQ) and acceptable measurement precision, further supporting the importance of condition-specific tools for evaluating functional limitations in this population [13].

The paired t-test results further validated these observations, demonstrating significant reductions in the Hand20 scores over time. This outcome is consistent with the findings of previous studies that reported notable postoperative improvements in Hand20 scores assessed using paired t-tests, underscoring the tool's sensitivity in detecting clinical changes [5]. Furthermore, the larger mean difference observed at six months compared to three months reflects progressive enhancements in hand function and symptom severity. Previous studies have similarly highlighted the Hand20 questionnaire's superior responsiveness compared to the Disability of the Arm, Shoulder, and Hand-Japanese Society for Surgery of the Hand (DASH-JSSH), confirming its effectiveness in capturing postoperative improvements [14]. These results are consistent with prior research supporting the efficacy of conservative management strategies for CMC joint osteoarthritis, further reinforcing the clinical relevance of targeted hand therapy interventions [15,16]. The statistically significant mean differences observed at three months (14.73, 95% CI: 4.99-24.46; p = 0.005) and six months (17.58, 95% CI: 8.82-26.33; p < 0.001) underscore the robustness and reliability of these findings. Collectively, these data emphasize the utility of the Hand20 as a sensitive and reliable tool for monitoring clinical changes over time, offering valuable insights into the sustained benefits of conservative management strategies for thumb CMC joint osteoarthritis.

From a clinical perspective, Hand20 offers several advantages. Its ease of administration and comprehensiveness make it a practical tool for clinicians and researchers. Previous studies have shown that the Hand20 questionnaire combines simplicity and broad applicability, making it highly acceptable, even for older patients [4]. Moreover, the Hand20 has demonstrated high reliability and responsiveness in clinical populations, with features that enhance its usability even among older adults [5]. The ability to detect moderate to large changes in hand function over time is especially valuable for evaluating the effectiveness of therapeutic interventions, guiding treatment decisions, and facilitating patient-centered care.

Although these results are promising, it is important to acknowledge that this study focused on a relatively small sample of 20 patients. Nevertheless, previous evaluations of Hand10, a shorter version of Hand20, have also demonstrated strong responsiveness in similarly small clinical populations [11]. Furthermore, the limited sample size may have affected the statistical power to detect small effects and resulted in wider confidence intervals, particularly in the reliability estimates. These limitations highlight the need for future studies with larger and more diverse populations to validate the findings and enhance generalizability. While the sample size is limited, the consistency of our findings with previous PROM validation studies provides preliminary support for the clinical utility of Hand20. Although these findings provide a strong foundation, further studies with larger cohorts are necessary to confirm the generalizability of these results. Similar validation studies with adequate sample sizes have already been successfully conducted for other upper-extremity questionnaires, such as the QuickDASH-JSSH [17]. These efforts highlight the importance of large-scale studies in establishing the reliability, validity, and responsiveness of PROMs in diverse clinical populations.

This study had several limitations that must also be considered. First, the retrospective design of the study may introduce potential biases, including variations in baseline characteristics and treatment adherence. Second, although the Hand20 questionnaire is validated for upper extremity disorders, it may not fully capture thumb-specific impairments, such as difficulties with precision grip, thumb-index opposition, or other fine motor tasks. Additionally, the small sample size typical of pilot studies may limit statistical power and the generalizability of findings. The absence of detailed demographic and clinical background data also limits the interpretability of subgroup characteristics. This study was also limited by its single-center design, which may limit external validity. Although the test-retest reliability was acceptable (ICC = 0.672), it falls in the moderate range and should be interpreted accordingly. Finally, the sample was gender-imbalanced, which may affect the generalizability of findings across sexes.

Future research should address these limitations by integrating additional PROMs or modifying existing tools to better capture the unique functional demands associated with thumb CMC joint osteoarthritis. Prospective study designs with larger sample sizes and more refined instruments are also warranted to confirm and extend these findings.

## Conclusions

This pilot study demonstrated that the Hand20 questionnaire is a moderately reliable and highly responsive instrument for evaluating treatment outcomes in patients with thumb CMC joint osteoarthritis. While the findings are promising, they should be interpreted with caution due to the small sample size and preliminary nature of the study. Nonetheless, the results support the potential clinical utility of Hand20 for monitoring disease progression and evaluating therapeutic efficacy. Future research should expand on these findings by examining its performance in larger, more diverse populations and exploring its applicability alongside other disease-specific measures.
